# High prevalence of antibiotic resistance in pathogenic foodborne bacteria isolated from bovine milk

**DOI:** 10.1038/s41598-022-07845-6

**Published:** 2022-03-09

**Authors:** Sima Hassani, Mir-Hassan Moosavy, Sahar Nouri Gharajalar, Seyed Amin Khatibi, Abolfazl Hajibemani, Zahra Barabadi

**Affiliations:** 1 Department of Food Hygiene and Aquatic, Faculty of Veterinary Medicine, University of Tabriz, P.O. Box: 5166614779, Tabriz, Iran; 2 Department of Pathobiology, Faculty of Veterinary Medicine, University of Tabriz, Tabriz, Iran; 3 Department of Clinical Sciences, Faculty of Veterinary Medicine, University of Tabriz, Tabriz, Iran; 4 Department of Tissue Engineering, School of Medicine, Hamadan University of Medical Sciences, Hamadan, Iran

**Keywords:** Antimicrobials, Bacteria

## Abstract

This study aimed to investigate the prevalence of foodborne pathogenic bacteria in bovine milk, their antibiogram phenotype, and the carriage of antibiotic resistance genes. Raw bovine milk samples (n = 100) were randomly collected from different suppliers in the northwest of Iran. Antibiotic-resistant patterns and the presence of antibiotic resistance genes were evaluated in the isolates. *Escherichia coli, Listeria monocytogenes*, *Staphylococcus aureus,* and *Salmonella* spp. were isolated from 78%, 47%, 25%, and 21% of samples, respectively. All isolates showed high rates of resistance to amoxicillin, penicillin, and cefalexin. The *bla*_TEM_ and *bla*_SHV_ genes were detected in 50.0% and 6.4% of *E. coli* isolates, respectively. Also, 28.5% and 19.0% of *Salmonella* isolates were positive for *bla*_TEM_ and *bla*_SHV_. The frequency of *mec*A and *bla*_Z_ in *S. aureus* isolates was 20.0% and 12.0%, respectively. The high prevalence of bovine milk contamination with antimicrobial-resistant species in this study necessitates precise control on antibiotic prescription in veterinary medicine.

## Introduction

The burden of foodborne diseases in humans remains largely unknown^1^. During the past decade, the incidence of foodborne microbial diseases has considerably increased in most countries^2^.

Milk and dairy products, as common food products in many countries, provide favorable environments for the growth of many microorganisms because of their nutrient composition^3^. Many studies have been performed to improve raw milk quality to reduce the risk of microbial contamination and to increase the chemical nutritional quality of dairy products^3,4^. In recent years, the consumption of raw milk has been increasingly welcomed due to its potential benefits such as having high nutritional content and beneficial bacteria as well as the prevention of lactose intolerance. However, due to the potential presence of pathogens and their toxins, the consumption of raw milk can pose a serious risk of foodborne disease to public health^5–8^. *Staphylococcus aureus, Salmonella* spp.*, Listeria monocytogenes,* and *Escherichia coli* are the most common pathogens that can be found in raw milk and dairy products made from raw milk^4,7^. Also, *S. aureus, L. monocytogenes*, and *Salmonella* spp. can contribute to bovine mastitis and be excreted directly into the milk^8–10^.

Inappropriate use of antibiotics is a common problem in medical and veterinary medicine, which may result in the development of multidrug-resistant microorganisms^11^. The antibiotic resistance in pathogenic bacteria is known as a big challenge for public health worldwide^12–14^. One of the most important enzymes involved in antibiotic resistance of bacteria is beta-lactamase, especially extended-spectrum beta-lactamase (ESBL), which deactivate the beta-lactam antibiotics through hydrolysis of beta-lactam ring^15^. The most common ESBL-producing genes are SHV (*bla*_SHV_), TEM (*bla*_TEM_), and CTX-M genes (*bla*_CTX-M_)^16^.

Over the last decades, *mec*A has been detected in *S. aureus*isolates^17^. The *mec*A gene is responsible for resistance to methicillin and other β-lactam antibiotics. This gene encodes a penicillin-binding protein (PBP2A) with a low affinity for β-lactam antibiotics^18,19^. Also, *bla*_Z_ has been reported as the main gene in *S. aureus* responsible for resistance against several antibiotics^20^. TEM and SHV-type β-lactamases are reported as the main causes of resistance in *E. coli*strains^21^. Also, numerous beta-lactamases such as TEM, SHV, PER, OXA, and CTX enzymes have been identified in different *Salmonella* species^22^. Therefore it seems important to investigate the antibiotic resistance patterns of pathogenic bacteria and the presence of associated encoding genes as the key elements of antibiotics resistance.

This study aimed to evaluate the prevalence of pathogenic foodborne bacteria in raw bovine milk through culture-based techniques, their antibiogram phenotype, and the presence of antibiotic resistance genes among the isolates using multiplex-PCR.

## Material and methods

### Sampling

Raw bovine milk samples (n = 100) were collected aseptically from different retail sellers in the northwest of Iran. At the seller level, all milk samples were stored in the refrigerator (≤ 4 °C). Samples were transported to the laboratory in an icebox at a temperature less than 4 °C. They were kept in a refrigerator at 4 ± 1 °C before analysis. The microbiological experiments were performed immediately. All microbiological culture mediums were provided by Merck Company (Darmstadt, Germany).

### Total bacterial count

Serial tenfold dilutions of raw milk samples were prepared using the tubes containing 9 ml of sterile % 0.1 peptone water (up to 1:10,000 dilutions)^23^. Then, 0.1 mL of each sample dilutions was cultured on Nutrient agar. The total mesophilic bacterial count was calculated after the plates were incubated aerobically at 37 °C for 48 h^24^.

### Isolation and detection of pathogenic bacteria

*Eecherichia coli* was isolated from samples according to the method of Feng et al.^25^and Ombarak et al.^26^ Three to five presumptive colonies (dark centered and flat colonies with metallic green sheen) from Levine’s Eosin Methylene Blue (L-EMB) agar plates were selected, transferred on tryptic soy agar (TSA), and incubated at 37 °C for 24 h. Biochemical confirmatory tests were performed according to the method of Feng et al.^25^ and Quinn et al.^27^.

*Staphylococcus aureus* was detected in the samples using Baird-parker agar. After incubation of plates at 37 °C for 48 h, typical black colonies with a clear zone were considered as presumptive *S. aureus*. The isolates were confirmed by biochemical tests such as coagulase, catalase, DNase, lecithinase, oxidase, Lysostaphin sensitivity, VP, urease, glucose, and mannitol fermentation^28^.

For isolation and detection of *L. monocytogenes,* samples were enriched in Buffered Listeria enrichment broth (BLEB) at 30 °C for 48 h. The bacterial suspension was streaked onto PALCAM agar and incubated at 35 °C for 48 h. The isolates were confirmed by motility test, gram staining, and biochemical tests such as catalase, oxidase, hemolysis, nitrate reduction, carbohydrate fermentation, Christie-Atkins-Munch-Peterson test (CAMP), methyl red, and Voges-Proskauer (MR/VP)^29^.

For isolation and detection of *Salmonella* spp*.,* the raw milk samples were cultured on Bismuth Sulphite agar (BSA), Brilliant Green, and Phenol-Red agar (BGA) for 24 h (BGA)/48 h (BSA) at 37 °C. The suspected colonies were transferred to Samonella-Shigella agar plates and incubated at 37 °C for another 24 h. The presumptive colonies on the plates were subjected to biochemical tests using Lysine Iron agar, Triple Sugar Iron Agar, Sulfide-Indole-Motility medium, and Christensen’s Urea agar^30^.

### Antimicrobial susceptibility test

Antibiotic susceptibility tests of isolates were performed by the Kirby-Bauer disk diffusion method according to the guidelines of clinical laboratory standards^31^. Isolates were included in the study based on isolation rank (time criterion). Based on this criterion, the first isolate of a particular species isolated from a single sample was included in the analysis^32^. Briefly, bacterial suspensions were prepared in tubes containing 0.9% (w/v) phosphate-buffered saline with turbidity adjusted to 0.5 McFarland standard. Using a sterile cotton swab, bacterial suspension was streaked uniformly on the surface of Muller-Hinton agar. Antibiotic disks (Padtan Teb, Iran) including amoxicillin (25 μg/disk), azithromycin (15 μg), penicillin (10 IU), cephalexin (30 μg), ceftriaxone (30 μg), gentamicin(10 μg), chloramphenicol (30 μg), and tetracycline (30 μg) were placed on the surface of cultures. The selected antimicrobials were representative of the major classes of antibiotics commonly used in veterinary and human medicine in Iran. Finally, the diameter of the inhibition zone around the disks was measured after incubation of plates at 37 °C for 24 h.

### Detection of *bla*_TEM_, *bla*_SHV_, *mec*A, and *bla*_Z_ genes using multiplex-PCR

The genomic DNA was extracted by boiling method^33^. The primers used for the detection of target genes are listed in Table 1. The reaction contents for each 25 μL PCR consisted of 5.5 μL of deionized water, 12.5 μL RED-Extract-N-Amp master mix 2 × (containing buffer, salts, dNTPs, Taq polymerase, REDTaq dye, and JumpStart Taq antibody) (Sigma-Aldrich, USA), 1 μL of each primer and 3 μL of template DNA. The PCR program for *bla*_TEM_ and *bla*_SHV_ genes included initial denaturation for 5 min at 94 °C followed by 32 cycles of denaturation at 94 °C for 30 s, annealing step at 54 °C for 30 s, extension step at 72 °C for 60 s, and a final extension step at 72 °C for 10 min. The PCR condition for *mec*A and *bla*_Z_ were as follows: initial denaturation at 95 °C for 4 min, 30 cycles of denaturation at 95 °C for 60 s, annealing step at 58 °C for 60 s, extension step at 72 °C for 60 sand final extension step at 72 °C for 4 min. PCR products were subjected to electrophoresis using 1.5% (w/v) agarose gel. The gel was stained with ethidium bromide. Ultraviolet transillumination (Biorad, USA) was applied for the visualization of DNA.

**Table 1:** PCR sets used for detection of target antibiotic-resistance genes in the selected foodborne bacterial isolated from bovine milk.

Genes	Primer sequence (5' → 3')	Accession number (GenBank)	Annealing (°C)	Amplicon size (bp)	References
*bla*_TEM_	F:ATC AGC AAT AAA CCA GC	NG_068216.1	54	516	Eid and Samir^34^
	R: CCC CGA AGA ACG TTT TC				
*bla*_SHV_	F: AGG ATT GAC TGC CTT TTTG	NG_068212.1	54	392	Yukawa et al.^35^
	R: ATT TGC TGA TTT CGCTCG				
*mec*A	F: AAA ATC GAT GGT AAA GGT TGG C	MK659556.1	58	532	Kim et al.^36^
	R: AGT TCT GCA GTA CCG GAT TTG C				
*bla*_Z_	F: TGA CCA CTT TTA TCA GCA ACC	MN689952.1	58	700	Meroni et al.^37^
	R: GCC ATT TCA ACA CCT TCT TTC				

## Results and discussion

Several studies have revealed that food products such as raw milk and dairy products made from raw milk may be the main sources for the outbreak of antibiotic-resistance pathogens which are known as a challenge for the safety of food products^38^. This problem is common in developing countries such as Iran, because of the poor food handling practices, inadequate food safety regulations, weak hygienic practices, insufficient financial resources to invest in food safety, weak regulatory systems, and inadequate education for food handlers. In the countries with outbreaks of foodborne diseases, the importance of pathogens like *S. aureus, E. coli*, *L. monocytogenes,* and* Salmonella* spp. has been reported as major causes^39^.

Numerous researchers previously reported the antimicrobial resistance of *E. coli* and *Salmonella* isolates from raw milk to the most common antibiotics in their studies^39–42^. Also, methicillin-resistant *S. aureus* as an emerging pathogen has become an important challenge for public health that has been isolated from raw milk^11,43^. The multidrug-resistant of *L*. *monocytogenes* isolates from raw milk to some commonly used antibiotics is reported in various countries such as Ethiopia^44^, Turkey^45^, Egypt^46^, and Pakistan^47^. So, the present study was designed to study the occurrence of the most common antibiotic-resistant foodborne pathogens from raw milk in Iran.

### Totalmesophilic bacterial count, isolation, and identification of bacterial species

The mean total mesophilic bacterial count of the examined raw milk samples in this study was 5.75 ± 0.85 log_10_ cfu mL^−1^ which was exceeded the permitted maximum value of raw milk contamination (5 log_10_ cfu mL^−1^)^48^. Our findings of the high rate of contamination in raw milk are in agreement with that of the previous study conducted in Tabriz, indicating the poor microbial quality of raw milk delivered to pasteurized milk plants^4^. In another study which was conducted in Allahabad city (India), the total bacterial count of examined milk samples was reported between 4.79 log_10_ cfu mL^−1^ by Yadav et al.^48^. Even, a higher level of contamination of about 6.32 ± 0.03 log_10_ cfu mL^−1^ was found for the raw milk samples from the collection centers of Guwahati city in India^49^. In general, the total bacterial count of more than 6 log_10_ cfu mL^−1^ reported by many countries is not desirable for raw milk supplies and is not usable for human consumption^50^.

The increased total bacterial count can be caused by the use of unsanitary equipment for milking, contamination of cow’s udders, inadequate cooling of milk, and occasionally by the milking of cows with mastitis^51^.

In the present study, 78% of samples were contaminated with *E. coli* with a mean count of 3.41 ± 0.41 log_10_ cfu mL^−1^. High rates of raw milk contamination with *E. coli* have been reported in many developing and developed countries. It has been reported that 90.67% of raw milk samples in Arusha, Tanzania were contaminated with *E. coli*^52^ as well as 76.4% of samples in Egypt^26^.

In our study, 25% of the raw milk samples were contaminated with *S. aureus* at an average level of 2.91 ± 0.80 log_10_ cfu mL^−1^. In agreement with our study, a study in California showed that 25.3% of the raw milk samples were contaminated with *S. aureus*^5^. In another study in Mansoura City, Egypt, the mean *S. aureus* counts were found to be 3.49 log_10_ cfu g^−1^ in raw milk samples^43^, and 70.4% of raw milk samples in Brazil were contaminated with *S. aureus*^53^. These results indicate the different quality of milk samples in different regions of the world.

According to the results of the present study, *L. monocytogenes* was isolated from 47% of the raw milk samples. Over 70% of positive samples contained *L. monocytogenes* at a level of less than 10 cfu ml^−1^. The mean count of this bacterium was detected at 0.60 ± 0.51 log_10_ cfu mL^−1^. Many studies in different countries reported the occurrence of *L. monocytogenes* by various rates of contamination in their raw milk supplies and related products. The occurrence of *L. monocytogenes* in raw milk has been reported in Kars city (Turkey)^45^. However, in research in Antakya, Turkey, *L. monocytogenes* was not detected in any of the raw milk samples^54^.

In the present study, *Salmonella* spp. was detected in 21% of the raw milk samples. After enrichment of samples followed by plating, the mean count of *Salmonella* spp. in the positive samples was detected at 0.26 ± 0.27 log_10_ cfu mL^−1^. Similar results have also been reported in different countries. The prevalence of *Salmonella* spp*.* in raw milk has also been reported in Arusha, Tanzania (37.33%)^52^, Egypt(44.44%)^55^, and Dhaka Metropolis, Bangladesh (25.71%)^56^.

### Antimicrobial susceptibility of isolates to the used antibiotics and detection of *bla*_TEM_,*bla*_SHV_, *mec*A, and *bla*_Z_ genes in the isolates

In this study, it was shown that all strains of *E. coli* were highly resistant to penicillin (88.46%), cefalexin (82.05%), and amoxicillin (70.51%) (Table 2). Fifty percent (50%) of *E. coli* isolates had *bla*_TEM_ and 6.41%of them were positive for *bla*_SHV_ (Table 3). Consistent with our study, another study reported that 83.1% of isolates of highly antibiotic-resistant *E. coli* strains, with 100% resistance to acetyl spiramycin, 100% to penicillin, 98.8% to lincomycin, 98.8% to oxacillin, 32.5% to cephalosporin, and 30.1% to ampicillin. The *bla*_TEM_ was the most frequently detected resistance gene (83.1%)^42^.

**Table 2:** Antibiotic resistance profile of *Escherichia coli* isolates (n = 78) from bovine milk samples.

Antimicrobial agent	Disk content	Interpretive categories and zone diameter breakpoints (nearest whole mm)*			No. of isolates (%)		
		R**	I	S	R	I	S
Azithromycin	15 μg	≤ 12	–	≥ 13	53 (67.94)	–	25 (32.05)
Chloramphenicol	30 μg	≤ 12	13–17	≥ 18	15 (19.23)	2 (2.56)	61 (78.20)
Ceftriaxone	30 μg	≤ 19	20–22	≥ 23	17 (21.79)	14 (17.94)	47 (60.25)
Penicillin	10 IU	≤ 14	–	≥ 15	69 (88.46)	–	9 (11.53)
Gentamicin	10 μg	≤ 12	13–14	≥ 15	6 (7.69)	3 (3.84)	69 (88.46)
Amoxicillin	25 μg	≤ 13	14–16	≥ 17	55 (70.51)	3 (3.84)	20 (25.64)
Tetracycline	30 μg	≤ 11	12–14	≥ 15	20 (25.64)	9 (11.53)	49 (62.82)
Cephalexin	30 μg	≤ 14	–	≥ 15	64 (82.05)	–	14 (17.94)

*From CLSI^31^.

**S susceptible, *I* intermediate, *R* resistant.

**Table 3:** Distribution of resistance genes in the selected foodborne bacterial isolated from bovine milk.

Target genes	No of isolates (%)			
	*E. coli*	*Salmonella* spp.	*L. monocytogenes*	*S. aureus*
*bla*_SHV_	5 (6.41%)	4 (19.04)	–	–
*bla*_Z_	–	–	–	3 (12.00)
*bla*_TEM_	39 (50%)	6 (28.57)	–	–
*mec*A	–	–	–	5 (20.00)

In the present study, *bla*_TEM_ was the most common resistance gene in *E. coli* isolates*.* However*,* only 50% of the resistant isolates to both penicillin and amoxicillin harbored this gene. Also, *bla*_SHV_ was present in five isolates of* E. coli.* All isolates containing this gene showed resistance to cephalexin, penicillin, and amoxicillin in phenotypic experiments.

The isolated strains of *L. monocytogenes* in our study were highly resistant to penicillin, cefalexin, and amoxicillin (97.87%) (Table 4). Since ampicillin is an important first-choice antibiotic for the treatment of listeriosis^57^, the isolates of *L. monocytogenes* were evaluated for the presence of known genes responsible for resistance to beta-lactam antibiotics (*bla*_TEM_, *bla*_SHV,_ *mec*A, *bla*_Z_) using the specific primers. However, none of the resistance genes were detected in *L. monocytogenes* (Table 3). Similar results were found by Marian et al.^58^ that showed 100% of *L. monocytogenes* strains in their study were resistant to ampicillin and penicillin, with no involvement of *bla*_Z_ and *mec*A genes in their resistance. Also, Bertsch et al.^57^ examined the antimicrobial susceptibility and antibiotic resistance genes in foodborne, clinical, and environmental isolates of *L. monocytogenes* that were negative for the presence of *bla*_Z_ and *mec*A genes.

**Table 4:** Antibiotic resistance profile of *Listeria monocytogenes* isolates (n = 47) from bovine milk samples.

Antimicrobial agent	Disk content	Interpretive categories and zone diameter breakpoints (nearest whole mm)*			No. of isolates (%)		
		R**	I	S	R	I	S
Azithromycin	15 μg	˂ 17	17–21	≥ 22	12 (25.53)	14 (29.78)	21 (44.68)
Chloramphenicol	30 μg	˂ 18	18–20	≥ 21	22 (46.80)	13 (27.65)	12 (25.53)
Ceftriaxone	30 μg	˂ 15	15–20	≥ 21	17 (36.17)	18 (38.29)	12 (25.53)
Penicillin	10 IU	< 8	8–28	≥ 29	46 (97.87)	0 (0)	1 (2.12)
Gentamicin	10 μg	< 18	18–20	≥ 21	24 (51.06)	21 (44.68)	2 (4.25)
Amoxicillin	25 μg	˂ 14	14–24	≥ 25	46 (97.87)	1 (2.12)	0 (0)
Tetracycline	30 μg	˂ 22	22–24	≥ 25	23 (48.93)	2 (4.25)	22 (46.80)
Cephalexin	30 μg	˂ 12	12–17	≥ 18	46 (97.87)	1 (2.12)	0 (0)

*From CA-SFM^59^, CLSI^31^, Hansen et al.^60^, and Soussy et al.^61^.

**S susceptible, *I* intermediate, *R* resistant.

The results of antimicrobial resistance tests showed that the isolated strains of Salmonella were highly resistant to penicillin (100%), cefalexin (100%), and amoxicillin (71.42%) (Table 5). High rates of antibiotic resistance for *Salmonella* spp. have been reported by many studies*.* In a study by Obaidat and Stringer (2019), more than 50% of *S. enterica* isolates in raw milk were resistant to kanamycin, streptomycin, amoxicillin, and tetracycline. In another study, the highest rate of antibiotic resistance for *Salmonella* was detected to ampicillin, chloramphenicol, streptomycin, sulfonamide, tetracycline, amoxicillin, ceftiofur, and ceftriaxone^41^. These results were consistent with the results obtained from the present study.

**Table 5:** Antibiotic resistance profile of *Salmonella* spp. isolates (n = 21) from bovine milk samples.

Antimicrobial agent	Disk content	Interpretive categories and zone diameter breakpoints (nearest whole mm)*			No. of isolates (%)		
		R**	I	S	R	I	S
Azithromycin	15 μg	≤ 12	–	≥ 13	8 (38.09)	–	13 (61.90)
Chloramphenicol	30 μg	≤ 12	13–17	≥ 18	6 (28.57)	1 (4.76)	14 (66.66)
Ceftriaxone	30 μg	≤ 19	20–22	≥ 23	5 (23.80)	3 (14.28)	13 (61.90)
Penicillin	10 IU	≤ 14	–	≥ 15	21 (100)	–	0 (0)
Gentamicin	10 μg	≤ 12	13–14	≥ 15	0 (0)	0 (0)	21 (100)
Amoxicillin	25 μg	≤ 13	14–16	≥ 17	15 (71.42)	3 (14.28)	3 (14.28)
Tetracycline	30 μg	≤ 11	12–14	≥ 15	5 (23.80)	7 (33.33)	9 (42.85)
Cephalexin	30 μg	≤ 14	–	≥ 15	21 (100)	–	0 (0)

*From CLSI^31^.

**S susceptible, *I* intermediate, *R* resistant.

In this study, six (28.57%) and 4 (19.04%) isolates of *Salmonella* spp*.* were positive for *bla*_TEM_ and *bla*_SHV_, respectively (Table 3). Four isolates with multidrug resistance to penicillin, ceftriaxone, amoxicillin, and cephalexin, carried both *bla*_TEM_ and *bla*_SHV_ genes. In a study by Ranjbar et al.^62^ the frequency of *Salmonella* spp. with *bla*_TEM_ and *bla*_SHV_ genes was 29.9% and 2.89%, while the prevalence of these two genes in Salmonella in another study was reported 15.38% and 12.82%, respectively^63^. The results of these studies were in agreement with the present study.

*Staphylococcus aureus* isolates were highly resistant to amoxicillin (100%), cephalexin (100%), and penicillin (84.00%), respectively (Table 6). Antimicrobial resistance in *S. aureus* species is very common in raw milk samples, as reported by many researchers. Li et al.^64^ indicated that 80.5% of *S. aureus* isolates were resistant to penicillin and ampicillin. The resistance of *S. aureus* isolates to penicillin G (87.9%), cloxacillin (75.9%), and amoxicillin (55.6%) was also reported by Al-Ashmawyet al.^43^ in Mansoura City, Egypt.

**Table 6:** Antibiotic resistance profile of* Staphylococcus aureus* isolates (n = 25) from bovine milk samples.

Antimicrobial agent	Disk content	Interpretive categories and zone diameter breakpoints (nearest whole mm)*			No. of isolates (%)		
		R**	I	S	R	I	S
Azithromycin	15 μg	≤ 13	14–17	≥ 18	8 (32.00)	8 (32.00)	9 (36.00)
Chloramphenicol	30 μg	≤ 12	13–17	≥ 18	6 (24.00)	13 (52.00)	6 (24.00)
Ceftriaxone	30 μg	≤ 13	14–20	≥ 21	6 (24.00)	12 (48.00)	7 (28.00)
Penicillin	10 IU	≤ 28	–	≥ 29	21 (84.00)	–	4 (16.00)
Gentamicin	10 μg	≤ 12	13–14	≥ 15	3 (12.00)	21 (84.00)	1 (4.00)
Amoxicillin	25 μg	≤ 28	–	≥ 29	25 (100)	–	0 (0)
Tetracycline	30 μg	≤ 14	15–18	≥ 19	7 (28.00)	11 (44.00)	7 (28.00)
Cephalexin	30 μg	≤ 21	–	≥ 22	25 (100)	–	0 (0)

*From CA-SFM^59^, CLSI^31^.

**S susceptible, *I* intermediate, *R* resistant.

**Figure 1:**
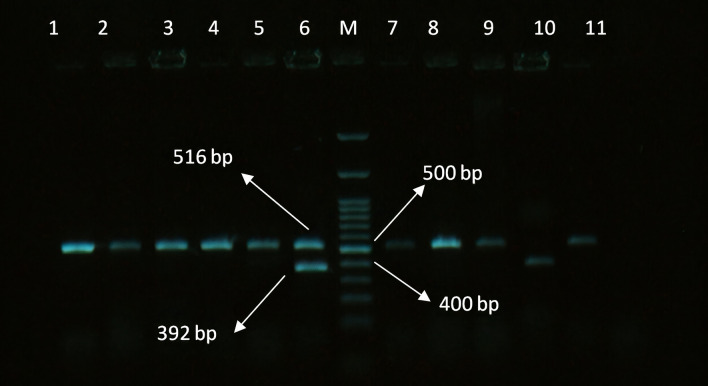
Electrophoresis pattern obtained by multiplex PCR for detection of *bla*_TEM_ and *bla*_SHV_ genes in *E. coli* isolates. M: marker 100 bp; lane 1, 2, 3, 4, 5, 6, 7, 8, 9, 11: amplification of *bla*_TEM_ gene at 516 bp; lane 6, 10: amplification of *bla*_SHV_ gene at 392 bp.

**Figure 2:**
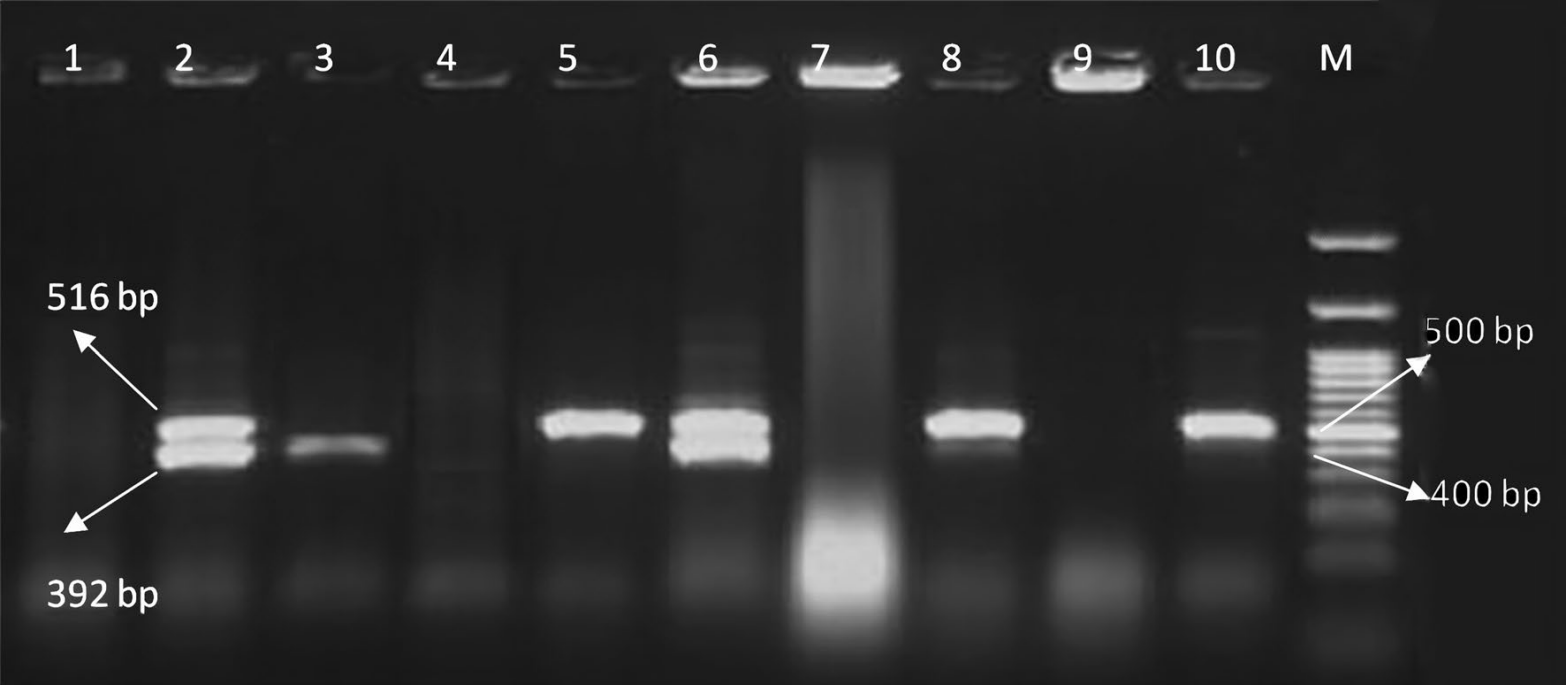
Electrophoresis pattern obtained by multiplex PCR for detection of *bla*_TEM_ and *bla*_SHV_ genes in *Salmonella* isolates. M: marker 100 bp; Lanes 2, 5, 6, 8, and 10: amplification of *bla*_TEM_ gene at 516 bp; lane 2, 3, 6: amplification of *bla*_SHV_ gene at 392 bp.

**Figure 3:**
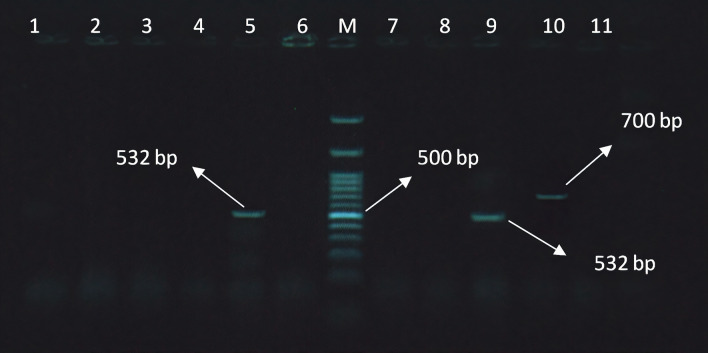
Electrophoresis pattern obtained by multiplex PCR for detection of *mec*A and *bla*Z genes in *S. aureus* isolates. M: marker 100 bp; Lanes 5 and 9: amplification of *mec*A gene at 532 bp; lane 10: amplification of *bla*_Z_gene at 700 bp.

The presence of *the mec*A gene was found in five (20%) isolates of *S. aureus* and the *bla*_Z_ gene was positive in three (12.00%) isolates of *S. aureus* (Table 3). Notably, *S. aureus* isolates with phenotypic resistances to penicillin, amoxicillin, ceftriaxone, and cephalexin always harbored *mec*A and *bla*_Z_ either individually or concurrently. These two genes are common genes involved in the antibiotic resistance of *S. aureus* strains. The electrophoresis pattern of the PCR products of the resistance genes in the bacteria under this study is shown in Figs. 1, 2 and 3.

The *bla*_Z_ and *mec*A were identified as resistance genes in *S. aureus* isolated from subclinical mastitis in Egypt^65^. In another study that investigated the genomic profile of *S. aureus* isolates from bulk tank milk and dairy cows with clinical mastitis, the prevalence of *bla*_Z_ gene was detected in 17.2% of isolates^66^.

Resistance to penicillin, amoxicillin, cephalexin, and ceftriaxone was more prevalent than the associated antibiotic resistance genes between isolates. The discrepancies between the phenotypic resistances and associated resistance genes in this study may be due to the fact that the entire suite of resistant genes, which could result in phenotypic resistance, was not evaluated in this study. Also, it is possible that the antibiotic-resistant genes detected may be mutated and/or non-functional, inducible or not expressed. Other mechanisms of resistance such as multidrug efflux pumps, mutations in outer membrane porins, or other unknown resistance genes may be involved in the phenotypic resistance^67,68^.

In the present study, high resistance levels and multidrug resistances against up to 7 antibiotics were detected between the evaluated isolates, with a high proportion for beta-lactams. Since beta-lactams are the most commonly used antibiotics in veterinary medicine, the emergence of beta-lactam-resistant pathogenic bacteria can be a serious threat to the wide use of these drugs^69^.

The occurrence of antibiotic-resistance pathogens in raw milk can be directly affected by farm management and practices. Regular cleaning of the farm can decrease the prevalence of antibiotic resistance pathogens^70^. The types of animal breeding (intensive, semi-intensive, or free-ranging) can influence the occurrence of antibiotic resistance pathogens due to the inappropriate administration of antibiotics. Excessive use of antibiotics in therapeutic and sub-therapeutic levels in dairy cattle farms can result in the presence of antibiotic-resistant pathogens in raw milk. So, if raw milk is not heat-treated, the presence of antibiotic-resistant foodborne pathogens in raw milk may pose food safety hazards to humans^70,71^.

## Conclusion

Our results show that raw milk has a great potential for transmission of antibiotic-resistant pathogens such as *E. coli, S. aureus, L. monocytogenes,* and *Salmonella* spp. In the present study, high levels of resistance were observed among the screened isolates to the most common beta-lactams such as amoxicillin, penicillin, and cefalexin. Also, the prevalence of beta-lactamase genes in *E. coli, S. aureus,* and *Salmonella* spp. provided evidence on the high risk of resistant food-borne pathogens to humans through raw milk.

Since antibiotics have extensive applications in dairy cattle farms in developing countries such as Iran; the microbiota of raw milk may contain relatively high levels of antibiotic-resistance bacteria. Therefore, enhancing the safety of milk and implementing good manufacturing practices are extremely important for the health of consumers. Pasteurization of raw milk, prevention of cross-contamination, storage of raw milk in cold temperature, appropriate authority supervision, and regulatory monitoring on the use of antibiotics in dairy cattle farms are necessary to ensure the safety of milk and dairy products.

The main route for the contamination of raw milk with resistant bacteria can be the subject of future studies to determine whether these bacteria get into the milk via cow’s udder or mixed into the milk during or after milking. Since phylogenetic assays can be used to ensure the genetic variations of resistant bacteria; it is recommended that these assays be performed on foodborne pathogenic isolates in future studies.

## Data Availability

The datasets generated during and/or analyzed during the current study are available from the corresponding author on reasonable request.

## References

[1] Newell DG, Koopmans M, Verhoef L, Duizer E, Aidara-Kane A, Sprong H, Opsteegh M, Langelaar M, Threfall J, Scheutz F, Van der Giessen J (2010). Food-borne diseases: The challenges of 20 years ago still persist while new ones continue to emerge. Int. J. Food Microbiol..

[2] World Health Organization. *General Information Related to Microbiological Risks in Food*. http://www.who.int/foodsafety/micro/general/en/index.html (2012)

[3] Moosavy MH, Kordasht HK, Khatibi SA, Sohrabi H (2019). Assessment of the chemical adulteration and hygienic quality of raw cow milk in the northwest of Iran. Qual. Assur. Saf. Crops Foods.

[4] Moosavy M, Mahmoudi R, Ghorbanpour E, Khatibi SA (2018). Evaluation of microbial and physicochemical characteristics of raw cow milk delivered to pasteurized milk plants in Tabriz city, Iran. J. Food Res..

[5] Heidinger JC, Winter CK, Cullor JS (2009). Quantitative microbial risk assessment for *Staphylococcus aureus* and Staphylococcus enterotoxin A in raw milk. J. Food Prot..

[6] Moosavy MH, Hallaj Salahipor M, Mostafavi E, Khatibi SA (2018). Risk factors for human brucellosis in Mianeh, Iran. J. Zoonotic Dis..

[7] Sugrue I, Tobin C, Ross RP, Stanton C, Hill C, Nero LA, De Carvalho AF (2019). Foodborne Pathogens and Zoonotic Diseases.

[8] Oliver SP, Jayarao BM, Almeida RA (2005). Foodborne pathogens in milk and the dairy farm environment: Food safety and public health implication. Foodborne Pathogen Disease.

[9] Ding T, Suo Y, Zhang Z, Liu D, Ye X, Chen S, Zhao YA (2017). Multiplex RT-PCR assay for *S. aureus*, *L. monocytogenes*, and *Salmonella* spp. detection in raw milk with pre-enrichment. Front. Microbiol..

[10] Cobirka M, Tancin V, Slama P (2020). Epidemiology and classification of mastitis. Animals.

[11] Asiimwe BB, Baldan R, Trovato A, Cirillo DM (2017). Prevalence and molecular characteristics of *Staphylococcus aureus*, including methicillin-resistant strains, isolated from bulk can milk and raw milk products in pastoral communities of South-West Uganda. BMC Infect. Dis..

[12] Frieri M, Kumar K, Boutin A (2017). Antibiotic resistance. J. Infect. Public Health.

[13] Aslam B, Wang W, Arshad MI, Khurshid M, Muzammil S, Rasool MH, Nisar MA, Alvi RF, Aslam MA, Qamar MU, Salamat MKF, Baloch Z (2018). Antibiotic resistance: A rundown of a global crisis. Infect. Drug Resist..

[14] Abadi ATB, Rizvanov AA, Haertlé T, Blatt NL (2019). World Health Organization report: Current crisis of antibiotic resistance. BioNano Sci..

[15] Thenmozhi S, Moorthy K, Sureshkumar B, Suresh M (2014). Antibiotic resistance mechanism of ESBL producing Enterobacteriaceae in clinical field: A review. Int. J. Pure Appl. Biosci..

[16] Livermore DM, Canton R, Gniadkowski M, Nordmann P, Rossolini GM, Arlet G, Ayala J, Coque TM, Kern-Zdanowicz I, Luzzaro F (2007). CTX-M: Changing the face of ESBLs in Europe. J. Antimicrob. Chemother..

[17] Strommenger B, Kettlitz C, Werner G, Witte W (2003). Multiplex PCR assay for simultaneous detection of nine clinically relevant antibiotic resistance genes in *Staphylococcus aureus*. J. Clin. Microbiol..

[18] Liao X, Cullen PJ, Liu D, Muhammad AI, Chen S, Ye X, Wang J, Ding T (2018). Combating *Staphylococcus aureus* and its methicillin resistance gene (*mec*A) with cold plasma. Sci. Total Environ..

[19] Elhassan MM, Ozbak HA, Hemeg HA, Elmekki MA, Ahmed LM (2015). Absence of the *mec*A gene in methicillin resistant *Staphylococcus aureus* isolated from different clinical specimens in Shendi City, Sudan. BioMed Res. Int..

[20] Olsen JE, Christensen H, Aarestrup FM (2006). Diversity and evolution of blaZ from *Staphylococcus aureus* and coagulase-negative *staphylococci*. J. Antimicrob. Chemother..

[21] Elumalai S, Muthu G, Selvam REM, Ramesh S (2014). Detection of TEM, SHV and CTX-M-type β-lactamase production among clinical isolates of *Salmonella* species. J. Med. Microbiol..

[22] Na SH, Moon DC, Kang HY, Song HJ, Kim SJ, Choi JH, Yoon JW, Yoon SS, Lim SK (2020). Molecular characteristics of extended-spectrum β-lactamase/AmpC-producing *Salmonella enterica* serovar Virchow isolated from food-producing animals during 2010–2017 in South Korea. Int. J. Food Microbiol..

[23] Weldaragay H, Yilma Z, Tekle-Giorgis Y (2012). Hygienic practices and microbiological quality of raw milk produced under different farm size in Hawassa, southern Ethiopia. Wudpecker J. Agric. Res. Rev..

[24] Lianou DT, Michael CK, Vasileiou NG, Petinaki E, Cripps PJ, Tsilipounidaki K, Katsafadou AI, Politis AP, Kordalis NG, Ioannidi KS, Gougoulis DA (2021). Extensive countrywide field investigation of somatic cell counts and total bacterial counts in bulk-tank raw milk in sheep flocks in Greece. Foods.

[25] Feng, P., Weagant, SD., Grant, M. A., Burkhardt, W., Shellfish, M. & Water, B. *BAM: Enumeration of Escherichia coli and the Coliform Bacteria*. https://www.fda.gov/food/laboratory-methods-food/bam-chapter-4-enumeration-Escherichia-coli-and-coliform-bacteria (2002).

[26] Ombarak RA, Hinenoya A, Awasthi SP, Iguchi A, Shima A, Elbagory ARM, Yamasaki S (2016). Prevalence and pathogenic potential of *Escherichia coli* isolates from raw milk and raw milk cheese in Egypt. Int. J. Food Microbiol..

[27] Quinn PJ, Markey BK, Leonard FC, Fitzpatrick ES, Fanning S, Hartigan PJ, Sayers M (2011). Veterinary Microbiology and Microbial Disease.

[28] Tallent, S., Hait, J., Bennett, R. W. & Lancette, G. A. *Bam Chapter12: Staphylococcus aureus*. https://www.fda.gov/food/laboratory-methods-food/bam-chapter-12-Staphylococcus-aureus (2019).

[29] Hitchins, A. D., Jinneman, K. & Chen, Y. *BAM Chapter 10: Detection of Listeria Monocytogenes in Foods and Environmental Samples, and Enumeration of Listeria Monocytogenes in Foods*. https://www.fda.gov/food/laboratory-methods-food/bam-chapter-10-detection-Listeria-monocytogenes-foods-and-environmental-samples-and-enumeration (2017).

[30] Andrews WH, Flowers RS, Siliker J, Bailey JS, Labbe RG, Downes FP, Ito K (2001). Compendium of Methods for the Microbiological Examination of Foods.

[31] Clinical and Laboratory Standards Institute. *Performance Standards for Antimicrobial Susceptibility Testing. CLSI Supplement M100 (31th ed.)*. https://clsi.org/standards/products/microbiology/documents/m100 (2021).

[32] Cornaglia G, Hryniewicz W, Jarlier V, Kahlmeter G, Mittermayer H, Stratchounski L, Baquero F (2004). European recommendations for antimicrobial resistance surveillance. Clin. Microbiol. Infect..

[33] Nayak R, Stewart TM, Nawaz MS (2005). PCR identification of *Campylobacter coli* and *Campylobacter jejuni* by partial sequencing of virulence genes. Mol. Cell. Probes.

[34] Eid S, Samir AH (2019). Extended-spectrum beta-lactamase and class 1 integrons in multidrug-resistant *Escherichia coli* isolated from turkeys. Vet. World.

[35] Yukawa S, Uchida I, Tamura Y, Ohshima S, Hasegawa T (2019). Characterisation of antibiotic resistance of *Salmonella* isolated from dog treats in Japan. Epidemiol. Infect..

[36] Kim YH, Kim HS, Kim S, Kim M, Kwak HS (2020). Prevalence and characteristics of antimicrobial-resistant *Staphylococcus aureus* and methicillin-resistant *Staphylococcus aureus* from retail meat in Korea. Food Sci. Anim. Resour..

[37] Meroni G, Soares Filipe JF, Drago L, Martino PA (2019). Investigation on antibiotic-resistance, biofilm formation and virulence factors in multi drug resistant and non multi drug resistant *Staphylococcus pseudintermedius*. Microorganisms.

[38] Ulusoy BH, Chirkena K (2019). Two perspectives of *Listeria monocytogenes* hazards in dairy products: The prevalence and the antibiotic resistance. Food Qual. Saf..

[39] Tadesse HA, Gidey NB, Workelule K, Hailu H, Gidey S, Bsrat A, Taddele H (2018). Antimicrobial resistance profile of *E. coli* isolated from raw cow milk and fresh fruit juice in Mekelle. Tigray. Ethiopia. Vet. Med. Int..

[40] Obaidat MM, Stringer AP (2019). Prevalence, molecular characterization, and antimicrobial resistance profiles of *Listeria monocytogenes*, *Salmonella enterica*, and *Escherichia coli* O157:H7 on dairy cattle farms in Jordan. J. Dairy Sci..

[41] Van Kessel JS, Sonnier J, Zhao S, Karns JS (2013). Antimicrobial resistance of *Salmonella enterica* isolates from bulk tank milk and milk filters in the United States†. J. Food Prot..

[42] Yu ZN, Wang J, Ho H, Wang YT, Huang SN, Han RW (2020). Prevalence, antimicrobial-resistance phenotypes and genotypes of *Escherichia coli* isolated from raw milk samples from mastitis cases in four regions of China. J. Glob. Antimicrob. Resist..

[43] Al-Ashmawy MA, Sallam KI, Abd-Elghany SM, Elhadidy M, Tamura T (2016). Prevalence, molecular characterization, and antimicrobial susceptibility of methicillin-resistant *Staphylococcus aureus* isolated from milk and dairy products. Foodborne Pathog. Dis..

[44] Girma Y, Abebe B (2018). Isolation, identification and antimicrobial susceptibility of *Listeria* species from raw bovine milk in Debre-Birhan Town, Ethiopia. J. Zoonotic Dis. Public Health.

[45] Aksoy A, Sezer Ç, Vatansever L, Gülbaz G (2018). Presence and antibiotic resistance of *Listeria monocytogenes* in raw milk and dairy products. Kafkas Univ. Vet. Fak. Dergis.

[46] Tahoun AB, Abou Elez RM, Abdelfatah EN, Elsohaby I, El-Gedawy AA, Elmoslemany AM (2017). *Listeria monocytogenes* in raw milk, milking equipment and dairy workers: Molecular characterization and antimicrobial resistance patterns. J. Glob. Antimicrob. Resist..

[47] Gohar S, Abbas G, Sarfraz M, Ali S, Ashraf M, Aslam R, Yaseen K (2017). Prevalence and antimicrobial resistance of *Listeria monocytogenes* isolated from raw milk and dairy products. Matrix Sci. Med..

[48] Yadav J, Paul S, Peter JK, Kumar Y, Singh AK, Masih F, Masih H (2014). Comparative evaluation of pathogenic bacterial incidence in raw and pasteurized milk. Int. J. Eng. Sci..

[49] Dinki N, Balcha E (2013). Detection of antibiotic residues and determination of microbial quality of raw milk from milk collection centres. Adv. Anim. Vet. Sci..

[50] Kivaria F, Noordhuizen J, Kapaga A (2006). Evaluation of the hygienic quality and associated public health hazards of raw milk marketed by smallholder dairy producers in the Dar es Salaam region, Tanzania. Trop. Anim. Health Prod..

[51] Vietoris V, Zajac P, Zubrická S, Čapla J, Čurlej J (2016). Comparison of total bacterial count (TBC) in bulk tank raw cow's milk and vending machine milk. Carpathian J. Food Sci. Technol..

[52] Lubote R, Shahada F, Matemu A (2014). Prevalence of *Salmonella* spp. and *Escherichia coli* in raw milk value chain in Arusha, Tanzania. Am. J. Res. Commun..

[53] Rall VLM, Vieira FP, Rall R, Vieitis RL, Fernandes A, Candeias JMG, Cardoso KFG, Araújo JP (2008). PCR detection of staphylococcal enterotoxin genes in *Staphylococcus aureus* strains isolated from raw and pasteurized milk. Vet. Microbiol..

[54] Aygun O, Pehlivanlar S (2006). *Listeria* spp. in the raw milk and dairy products in Antakya, Turkey. Food Control.

[55] Elafify M, Darwish WS, Al-Ashmawy M, Elsherbini M, Koseki S, Kawamura S, Abdelkhalek A (2019). Prevalence of *Salmonella* spp. in Egyptian dairy products: Molecular, antimicrobial profiles and a reduction trial using d-tryptophan. J. Consum. Prot. Food Saf..

[56] Yasmin S, Parveen S, Munna MS, Noor R (2015). Detection of *Salmonella* spp. and microbiological analysis of milk and milk based products available within Dhaka Metropolis, Bangladesh. Microbiol. Res. J. Int..

[57] Bertsch D, Muelli M, Weller M, Uruty A, Lacroix C, Meile L (2014). Antimicrobial susceptibility and antibiotic resistance gene transfer analysis of foodborne, clinical, and environmental Listeria spp isolates including *Listeria monocytogenes*. Microbiol. Open.

[58] Marian M, Aminah SS, Zuraini M, Son R, Maimunah M, Lee H, Wong W, Elexson N (2012). MPN-PCR detection and antimicrobial resistance of *Listeria monocytogenes* isolated from raw and ready-to-eat foods in Malaysia. Food Control.

[59] CA-SFM (2003). Comitéde l’Antibiogramme de la Socie´te´ Franc¸aise de Microbiologie Report 2003. Int. J. Antimicrob. Agents.

[60] Hansen JM, Gerner-Smidt P, Bruun B (2005). Antibiotic susceptibility of *Listeria monocytogenes* in Denmark 1958–2001. APMIS.

[61] Soussy CJ, Cluzel R, Courvalin P (1994). Definition and determination of in vitro antibiotic susceptibility breakpoints for bacteria in France. Eur. J. Clin. Microbiol. Infect. Dis..

[62] Ranjbar R, Ardashiri M, Samadi S, Afshar D (2018). Distribution of extended-spectrum β-lactamases (ESBLs) among *Salmonella* serogroups isolated from pediatric patients. Iran. J. Microbiol..

[63] Aljanaby AAJ, Medhat AR (2017). Research article prevalence of some antimicrobials resistance associated-genes in *Salmonella typhi* isolated from patients infected with typhoid fever. J. Biol. Sci..

[64] Li L, Zhou L, Wang L, Xue H, Zhao X (2015). Characterization of methicillin-resistant and-susceptible staphylococcal isolates from bovine milk in northwestern China. PLoS ONE.

[65] Abdeen EE, Walid M, Hussien H, Roshdy S (2015). PCR for detection of virulence and antibiotic resistance genes of coagulase-positive *Staphylococcus aureus* from clinical mastitis in Egypt. Int. J. Basic Appl. Sci..

[66] Ronco T, Klaas IC, Stegger M, Svennesen L, Astrup LB, Farre M, Pedersen K (2018). Genomic investigation of *Staphylococcus aureus* isolates from bulk tank milk and dairy cows with clinical mastitis. Vet. Microbiol..

[67] Davis MA, Besser TE, Orfe LH, Baker KN, Lanier AS, Broschat SL, New D, Call DR (2011). Genotypic-phenotypic discrepancies between antibiotic resistance characteristics of *Escherichia coli* isolates from calves in management settings with high and low antibiotic use. Appl. Environ. Microbiol..

[68] Smith M, Do TN, Gibson JS, Jordan D, Cobbold RN, Trott DJ (2014). Comparison of antimicrobial resistance phenotypes and genotypes in enterotoxigenic *Escherichia coli* isolated from Australian and Vietnamese pigs. J. Glob. Antimicrob. Resist..

[69] Ghazaei C (2019). Phenotypic and molecular detection of Beta-Lactamase enzyme produced by *Bacillus cereus* isolated from pasteurized and raw milk. J. Med. Bacteriol..

[70] Kamaruzzaman EA, Abdul Aziz S, Bitrus AA, Zakaria Z, Hassan L (2020). Occurrence and characteristics of extended-spectrum β-Lactamase-producing *Escherichia coli* from dairy cattle, milk, and farm environments in peninsular Malaysia. Pathogens.

[71] Wallensten A, Hernandez J, Ardiles K, Gonzalez-Acuna D, Drobni M, Olsen B (2011). Extended spectrum beta-lactamases detected in *Escherichia coli* from gulls in Stockholm, Sweden. Infect. Ecol. Epidemiol..

